# Lactate Secretion by Monocytes as a Determinant of Innate Immune Cell Fitness in Healthy Elderly

**DOI:** 10.1111/acel.70220

**Published:** 2025-10-13

**Authors:** Lisa Smeehuijzen, Frank Vrieling, Jenny Jansen, Hendrik J. P. van der Zande, Thomas M. Houslay, Gabriele Gross, Janna A. van Diepen, Lydia A. Afman, Rinke Stienstra

**Affiliations:** ^1^ Division of Human Nutrition and Health Wageningen University Wageningen the Netherlands; ^2^ Reckitt/Mead Johnson Nutrition Institute Hull UK; ^3^ Reckitt/Mead Johnson Nutrition Institute Nijmegen the Netherlands; ^4^ Department of Internal Medicine (463) Radboud University Medical Center Nijmegen the Netherlands

## Abstract

Immune cell metabolism is increasingly recognized as an important regulator of immune function, but its role in age‐related immune dysfunction, chronic inflammation, and cardiometabolic complications in humans remains incompletely understood. This study investigated the impact of aging on monocyte metabolic and functional signatures in a healthy elderly population. We aimed to leverage these immunometabolic signatures to identify healthy elderly individuals with reduced immune cell fitness and, therefore, potentially at a higher risk for age‐related complications. We characterized lactate and cytokine secretion, phagocytic capacity, and glycolytic and oxidative metabolic responses in monocytes from 103 elderly individuals and included 52 young adults as a reference group with healthy immune responses. We observed strong similarities in monocyte functional and metabolic signatures between young adults and elderly individuals. However, monocytes from the elderly secreted significantly more cytokines and displayed more ATP‐linked respiration and a reduced proton leak compared to young adults. These significant differences were driven by a subgroup within the elderly population characterized by higher monocyte lactate secretion compared to the remainder of the elderly and young adults and were therefore classified as “immune‐unfit”. The immune‐unfit elderly exhibited “hyperactive” monocytes, evidenced by significantly higher metabolic and functional signatures. Interestingly, compared to immune‐fit individuals, immune‐unfit elderly individuals had significantly elevated levels of circulating vascular endothelial growth factor and low‐density lipoprotein cholesterol. Hence, we propose lactate secretion from monocytes as a parameter to classify “immune‐unfit” elderly individuals with divergent immunometabolic properties of monocytes that could reflect increased susceptibility to age‐related cardiometabolic complications.

**Trial Registration:** NCT05940337

## Graphical abstract

Lactate secretion by monocytes can be used to classify healthy elderly individuals with reduced immune fitness exemplified by hypermetabolic and hyperinflammatory responses. This classification may reflect increased susceptibility to cardiometabolic complications given its link with higher circulating LDL‐c and VEGF levels.

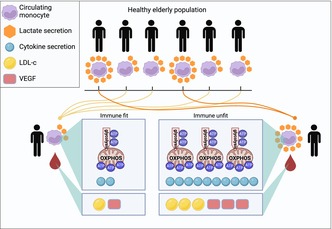

## 1 Introduction

Aging is associated with increased vulnerability to infection, elevated prevalence of chronic disease, impaired wound healing, and reduced vaccination efficiency (López‐Otín et al. 2013; Baechle et al. 2023). These age‐related complications are driven by immune dysfunction, which is characterized by chronic inflammation and inadequate immune responses (Baechle et al. 2023). Innate immune cells play an important role in chronic inflammation through elevated cytokine secretion and reduced activity of resolution mechanisms (Hearps et al. 2012; Albright et al. 2016). Furthermore, reduced Toll‐like receptor (TLR) expression and phagocytosis contribute to the elevated infection risk among the elderly (Solana et al. 2012). Hence, innate immune cells have been proposed as a potential target to reduce chronic inflammation in the elderly to reduce the risk for age‐related complications (De Maeyer and Chambers 2021).

Understanding the signatures of chronic inflammation in innate immune cells, including circulating monocytes, of elderly individuals will be crucial to understand the risk for age‐related complications. Cytokine secretion by innate immune cells is frequently reported, and while some studies report reduced cytokine secretion, most studies show elevated cytokine levels by innate immune cells from elderly compared to young individuals (Hearps et al. 2012; Lissner et al. 2015; Shchukina et al. 2021; Cao et al. 2022; Qian et al. 2012; Ong et al. 2018; Lee et al. 2014). Nonetheless, the underlying mechanisms of these functional alterations in innate immune cells are poorly understood. It is currently well‐recognized that the metabolism of immune cells is an important determinant of immune functionality (O'Neill et al. 2016). Activated immune cells upregulate metabolic pathways for the production of energy and biosynthetic precursors required for effector function, such as cytokine production. Furthermore, several metabolic enzymes directly or indirectly regulate inflammatory responses. For example, the glycolytic enzyme hexokinase 1 has been suggested to regulate NLRP3‐inflammasome activity, thereby regulating cytokine responses (Yu et al. 2022). Concurringly, reduced activity of the glycolytic pathway by 2‐deoxyglucose hampers macrophage activation (Pająk et al. 2024). Furthermore, mitochondrial dysfunction, exemplified by reduced mitochondrial biosynthesis, impaired mitophagy, and elevated ROS production, is commonly described in the aging process (Srivastava 2017; van Beek et al. 2019). Indeed, gene expression studies have shown reduced expression of mitochondrial electron‐transport chain genes in monocytes from healthy elderly, potentially indicating that impaired oxidative metabolism could be an early event in monocyte dysfunction (Shchukina et al. 2021; Saare et al. 2020). However, studies on metabolic signatures of innate immune cells from elderly individuals are scarce, and the role of immunometabolic signatures in age‐related immune dysfunction is currently unknown.

It has been shown that the immune system is highly variable among individuals, as evidenced by differences in immune cell populations and functional responses (Patin et al. 2018; Ter Horst et al. 2016). Such variation has also been observed among elderly individuals, who may differ greatly in terms of immune dysfunction and associated risk for age‐related complications despite being similar in age (Kaczorowski et al. 2017). Early identification of healthy elderly at risk for age‐related complications provides a window of opportunity for preventive strategies. Therefore, previous studies have laid the foundation for identifying individuals at risk for age‐related complications by computing an individual's immunological age (Sayed et al. 2021; Alpert et al. 2019). These immune age metrics estimate an individual's risk for age‐related disease based on circulating inflammatory markers and immunophenotyping. While altered immune phenotypes are likely driven by intracellular functional and metabolic alterations, immunophenotyping studies merely provide a proxy for functional and metabolic signatures of immune cells. Yet, insight into these signatures of circulating monocytes, which could represent a level of immune fitness, will be crucial given the interest in innate immune cells as therapeutic targets for preventive strategies against age‐related complications. Additionally, metabolic signatures of monocytes may serve as early markers for immune dysfunction since they may precede altered functional responses and phenotypic marker expression.

To enhance our understanding of the impact of aging on monocyte metabolism and functional responses, we comprehensively determined metabolic and functional signatures of monocytes from healthy elderly individuals and compared them to young adults that represent healthy immune responses of monocytes. These signatures were generated using ex vivo characterization of monocyte metabolic and functional responses using Seahorse assays, pathogenic challenges for lactate and cytokine secretion, and phagocytosis assays. Additionally, using young adults as a reference group, we aimed to exploit the monocyte immunometabolic signatures to define immune fitness. These signatures were then used for identifying healthy elderly individuals with reduced immune fitness and a potentially elevated risk for age‐related inflammatory complications, enabling us to intervene with prevention strategies at an early stage with a larger window of opportunity for risk reduction.

## 2 Methods

### 2.1 Study Participants

This study was approved by the medical ethics committee at Wageningen University (NL70696.081.19) and registered in ClinicalTrials.gov under the identifier NCT05940337. Study participants were recruited around Wageningen University, The Netherlands, between September 2020 and December 2021. We screened 278 subjects for eligibility based on criteria for good general health, meaning absent diagnosis for any disease and no use of medication with effects on the immune system. Additionally, BMI limits for the young adult group were set to 18–25 kg/m^2^ and 20–30 kg/m^2^ for the elderly group. The complete list of exclusion criteria is available in Table S1. The screening process included 53 healthy young adults aged between 20 and 30 years and 106 healthy elderly individuals aged between 60 and 75 years. Dropouts were attributed to CRP blood levels repeatedly exceeding 10 mg/L on > 2 days (*N* = 1), fainting during blood collection (*N* = 1), abnormal high lymphocyte counts (*N* = 1), or personal reasons unrelated to the study (*N* = 1). Consequently, the study retained *N* = 52 young adults and *N* = 103 elderly participants.

### 2.2 Study Design

The study had a cross‐sectional design. On the evening preceding the study visit, participants consumed a standardized meal ad libitum (per 100 g: 476 kJ 22 energy% protein, 24 energy% fat, and 51 energy% carbohydrate). This meal was consumed no later than 8:00 p.m. After the meal, participants were restricted from consuming anything except water. The next morning, participants visited the human research facility at Wageningen University to collect a fasted blood sample. Before blood collection, a CRP level ≤ 10 mg/L was confirmed through a finger prick (QuickRead CRP test, Orion Diagnostica Oy). Additionally, body height and body weight were measured in duplicates. Fat mass, lean mass, and bone mineral density were measured by dual‐energy X‐ray absorptiometry (DXA).

### 2.3 Monocyte Purification

150 mL of blood samples collected in EDTA tubes (367864, Becton Dickinson) was transferred to Leucosep tubes (227290P, Greiner Bio‐one) containing Ficoll‐Plaque PLUS (17144003, Cytivia) for density‐gradient purification for peripheral blood mononuclear cells (PBMCs). Monocytes were purified from PBMCs by magnetic‐activated cell sorting using CD14+ microbeads (130–050‐201, Miltenyi Biotec). Freshly isolated monocytes were directly used for functional and metabolic characterization.

### 2.4 Monocyte Pathogenic Activation

Monocytes were suspended in monocyte growth medium (RPMI1640, 21870076, Gibco) supplemented with 2 mM glutaMAX (35050061, Fisher Scientific) and 1% penicillin + streptomycin (P/S) (15323671, Fischer Scientific). They were seeded at a density of 0.5 × 10^6^ cells per well in 24‐well plates and incubated for 1 h at 37°C, 5% CO_2_. After 1 h, adherence was microscopically confirmed, and 2× lipopolysaccharide (LPS, final conc: 10 ng/mL, from *Escherichia coli* O55:B5, L6529, Merck), Pam_3_Cys‐ser‐lys_4_ (Pam3Cys, final conc: 10 ng/mL, L2000, EMC microcollections), or heat‐killed *Staphylococcus aureus* (HKSA, final conc: 1 × 10^6^/mL, prepared in‐house) were added and followed by 24 h incubation at 37°C, 5% CO_2_. After 24 h, the supernatant was collected and stored at −80°C until further analysis.

### 2.5 Phagocytosis Assay

Monocytes were seeded at a density of 0.2 × 10^6^/well in round‐bottom 96‐well plates and pre‐incubated for 30 min with either monocyte growth medium alone or supplemented with the metabolic inhibitors 2DG (22 mM) (D8375, Merck) and oligomycin (1 μM) (75351, Merck) to inhibit metabolic activity and thereby serve as a negative control for phagocytosis. After 30 min of pre‐incubation at 37°C 5% CO_2_, 1 μm green fluorescent microspheres (15702–10, Polysciences) in a bead‐to‐cell ratio of 10‐to‐1 were incubated for 1.5 h at 37°C 5% CO_2_. After incubation, cells were centrifuged and resuspended in 50 μL 3:1 PBS + Trypan blue (93595, Merck) to quench the signal of extracellular beads. The cells were subsequently acquired on a CytoFLEX S cytometer and analyzed within 30 min to prevent toxicity from Trypan Blue. Cells with phagocytic activity were identified by FITC+ Trypan Blue‐ and expressed as a percentage of the number of single cells.

### 2.6 Seahorse Assays

Monocytes were resuspended in monocyte growth medium and seeded in a density of 0.2 × 10^6^/well in 96‐well Seahorse plates (101085‐004, Agilent) in quintuplicate. Upon adherence, the medium was replaced by Seahorse base medium (RPMI pH 7.4, 103576–100, Agilent) supplemented with 2 mM L‐glutamine (G7512, Merck). For the mitochondrial stress test, the medium was additionally supplemented with 10 mM glucose (G8644, Merck). Monocytes were incubated in Seahorse base medium for 1 h in a CO_2_‐free environment at 37°C prior to the assay.

Oxygen consumption rates (OCR) and extracellular acidification rates (ECAR) were measured in a Seahorse XF96 Extracellular Flux Analyzer (Seahorse Bioscience, Agilent). After 5 baseline measurements, monocytes were sequentially exposed to (A) 11 mM glucose (3 cycles), (B) 10 μg/mL Pam3Cys (6 cycles), (C) 2.5 μM Antimycin (A8674, Merck) + 1.25 μM rotenone (R8875, Merck) (3 cycles), and (D) 22 mM 2DG (3 cycles), or to the mitochondrial stress test consisting of (A) 1 μM oligomycin (3 cycles), (B) 1 μM FCCP (C2920, Merck) + 1 mM pyruvate (11360070, Fisher Scientific) (6 cycles), and (C) Antimycin + rotenone (3 cycles).

#### 2.6.1 Seahorse: Data Pre‐Processing

We used our customized Python pipeline—previously described in Smeehuijzen et al. (2024)—for data compilation and pre‐processing. Briefly, negative values for ECAR and OCR were removed, and a replicate was removed if ≤ 40% of the data was retained. If this resulted in ≤ 2 replicates for a study participant, the participant was removed from the analysis. ECAR rates were corrected for post‐2DG acidification and OCR for post‐rotenone + antimycin oxygen consumption.

#### 2.6.2 Seahorse: Calculation of Variables

Variables of the glucose and Pam3Cys exposure assays were calculated as follows: baseline ECAR and OCR were calculated by the average of the last 3 of the 5 baseline cycles. The effect of glucose was calculated by the maximal ECAR or average OCR during the 3 measurement cycles upon glucose injection minus baseline ECAR or OCR, reflecting the glucose‐induced increase/decrease in ECAR or OCR relative to baseline. The effect of Pam3Cys was calculated by the maximal ECAR or OCR of 6 measurement cycles upon Pam3Cys injection minus the maximal ECAR or OCR upon glucose injection, reflecting the Pam3Cys‐induced ECAR or OCR relative to glucose‐induced ECAR or OCR. Finally, the metabolic potential was calculated as the maximal ECAR or OCR measured during the complete assay.

The mitochondrial stress test variables were calculated as follows: the baseline OCR was calculated by the average of the last 3 of the 5 baseline cycles. ATP‐linked respiration was calculated by subtracting the lowest OCR measured during the 3 cycles upon oligomycin injection from the baseline OCR. The proton leak was equal to the lowest OCR measured upon oligomycin injection, as all data were priorly corrected for post‐rotenone‐antimycin oxygen consumption. Hence, the maximal respiration was equal to the highest OCR measured during 6 cycles upon FCCP + pyruvate injection. The spare respiration was then calculated by subtracting baseline OCR from the maximal respiration.

### 2.7 Lactate Measurements

Lactate levels in diluted supernatants were quantified by fluorescence‐activated Amplex Red (A12222, Invitrogen) by horseradish peroxidase (31490, Fisher Scientific), relying on the generation of hydrogen peroxide facilitated by lactate oxidase (L9795, Merck). Lactate concentrations were determined using a lactate standard (L7022, Merck).

### 2.8 Cytokine Measurements

Secreted cytokines were determined in supernatants by sandwich enzyme‐linked immune‐sorbent assays (ELISA) for TNFα (R&D Systems, DY210), IL‐6 (R&D Systems, DY206), IL‐10 (R&D Systems, DY217B), IL‐1β (R&D Systems, DY201), and CXCL8 (R&D Systems, DY208) according to manufacturer's instructions.

### 2.9 Blood Measurements

Venous blood collected in EDTA tubes (367864, Becton Dickinson) was centrifuged for 10 min at 1200 × *g* at 4°C. Serum tubes were left for clotting for 30 min at room temperature and centrifuged for 10 min at 1300 × *g* at RT. Lithium‐heparin and sodium‐fluoride tubes were centrifuged for 10 min at 1300 × *g* at RT. Plasma and serum samples were aliquoted and snap‐frozen in liquid nitrogen and stored at −80°C until analysis. Plasma glucose, insulin, triglycerides, high‐density lipoprotein cholesterol (HDL‐c), and low‐density lipoprotein cholesterol (LDL‐c) were measured by the central laboratory of the hospital Gelderse Vallei, Ede, The Netherlands. The homeostatic model assessment for insulin resistance (HOMA‐IR) was calculated by fasting glucose (mmol/L) × fasting insulin (mU/L)/22.5 (Matthews et al. 1985).

Circulating inflammatory proteins were measured in plasma samples using the Olink target 96‐inflammatory protein panel by proximity extension assay (Olink, Uppsala, Zweden). Data quality checks were performed using Olink internal QC methods, and 16 subjects and 14 proteins were excluded based on a deviation of ≥ 0.3 normalized protein expression level from the median of control samples (pooled plasma samples). The levels of 78 inflammatory markers are expressed as arbitrary standardized units for 138 study subjects.

### 2.10 Classification of Immune Fitness

Immune fitness was determined based on the 95% confidence interval (CI) of basal monocyte lactate secretion in young adults. Elderly individuals with monocyte lactate secretion levels beyond the young adults' 95% CI were classified as immune unfit.

### 2.11 Data Analysis

Pairwise comparisons of subject characteristics between young adults and the elderly were analyzed by unpaired *T*‐tests or one‐way ANOVA with Tukey pairwise comparisons after stratification for immune fitness. Differences in categorical data were analyzed by Fisher's exact test. Between and within group differences in monocyte function and metabolism were analyzed by two‐way ANOVA with Tukey post hoc pairwise comparisons. Side‐by‐side comparisons of circulating proteins (Olink) between immune‐fit and immune‐unfit elderly and clinical data were performed using one‐way ANOVA with FDR correction in the case of the Olink analysis. PCA analysis was performed using the R package “factoextra” and PCA plots and histograms were created using “ggplot2”. Statistical analyses and data visualization were performed in Rstudio 2023.06.1 (R4.3.0) and GraphPad PRISM 10.0.3. Figures are created with biorender.com.

## 3 Results

### 3.1 Study Participants

**FIGURE 1:**
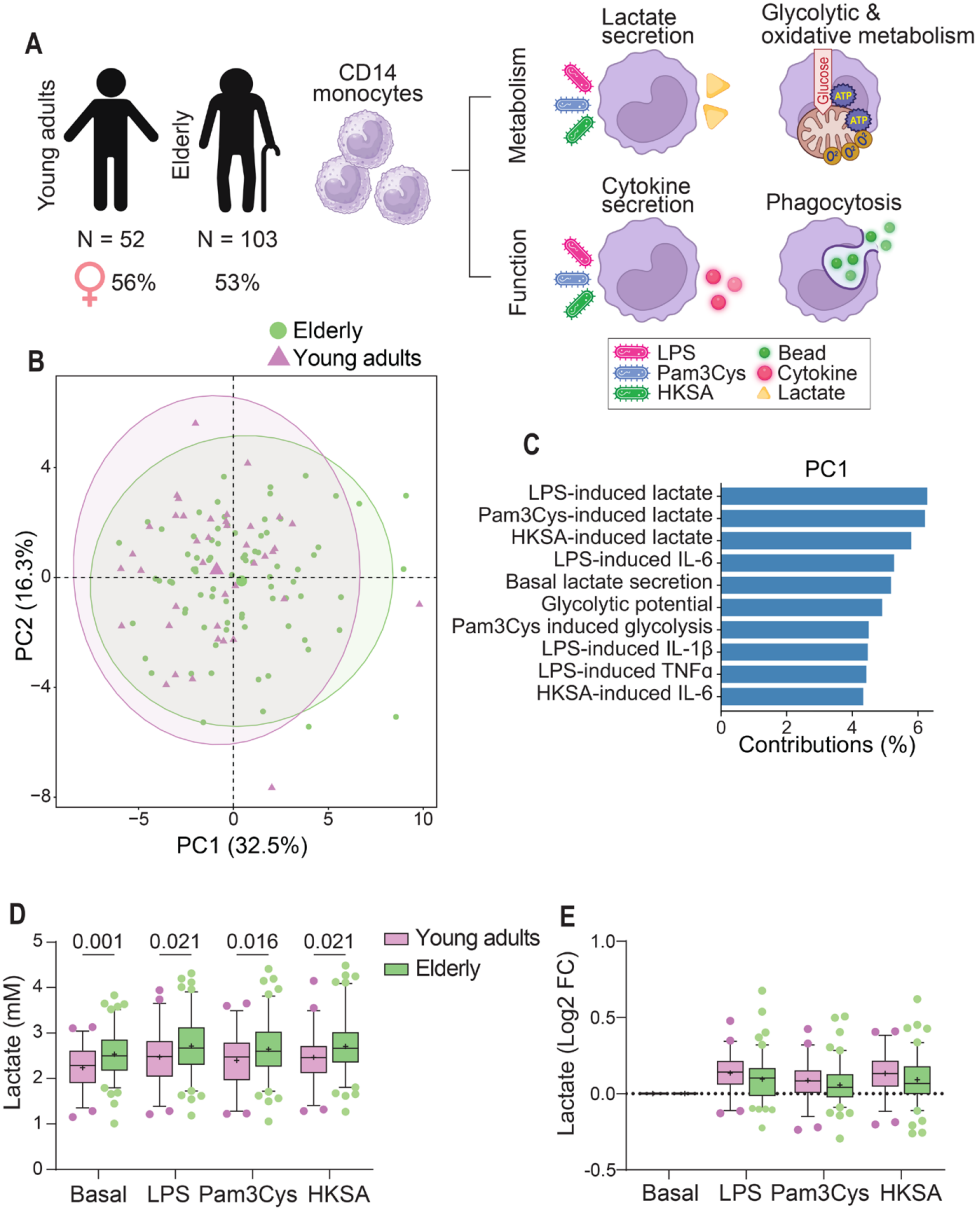
Monocyte immunometabolic signatures between young adults and elderly. (A) Freshly isolated monocytes from young adults and elderly individuals were used in ex vivo assays to determine functional and metabolic responses. (B) Principal component analysis on the variables for monocyte basal and activated functional and metabolic responses, including lactate secretion, glycolytic activity, oxidative metabolic activity, cytokine secretion, and phagocytosis. (C) Loading plot for the 10 highest contributing variables for principal component 1. (D) Lactate secretion levels in mM by monocytes upon 24 h stimulation to RPMI only (basal) and in the presence of LPS (10 ng/mL), Pam3Cys (10 ng/mL), or HKSA (1 × 10^6^/mL) measured in supernatants. (E) Log2 normalized to basal lactate secretion. Boxplots show median (middle line) and average (+). Whiskers cover 5%–95% of the data. Significance is reported in *p*‐values from unpaired *T*‐tests. HKSA, heat‐killed *Staphylococcus aureus* ; LPS, lipopolysaccharide.

We enrolled 52 young adults with a BMI ranging from 18.5 to 25 kg/m^2^ and 103 elderly individuals with a BMI between 20 and 30 kg/m^2^ (subject characteristics are listed in Table 1). All participants had no diagnosed diseases and were not using any medication that could directly impact the immune response (see Table S1 for the inclusion and exclusion criteria). Circulating monocytes were isolated and freshly used in functional and metabolic assays, including seahorse analysis and pathogenic stimulations, to define monocyte immunometabolic signatures (Figure 1A).

**TABLE 1:** Subject characteristics.

	Young adults	Elderly	*p*
	*N* = 52	*N* = 103	
Age (median years, (range))	23 (20–29)	69 (60–75)	≤ 0.001
Women (%)	56	53	0.696
Bodyweight (kg)	67.8 ± 10.1	72.9 ± 11.7	0.009
BMI (Kg/m^2^)	21.7 ± 1.8	24.3 ± 3.0	≤ 0.001
Waist circumference	76.2 ± 6.8	91.8 ± 10.0	≤ 0.001
Waist‐to‐hip ratio	0.89 ± 0.07	0.95 ± 0.07	≤ 0.001
Total body fat (%)	20.1 ± 7.6	28.0 ± 8.8	≤ 0.001
Lean mass (%)	75.5 ± 7.5	68.1 ± 8.5	≤ 0.001
Fasting glucose (mmol/L)	5.0 ± 0.4	5.4 ± 0.6	≤ 0.001
Fasting insulin (mU/L)	4.3 ± 2.4	6.0 ± 4.2	0.01
HOMA‐IR	1.0 ± 0.5	1.5 ± 1.1	0.002
Triglycerides (mmol/L)	1.0 ± 0.4	1.2 ± 0.4	0.018
Total cholesterol (mmol/L)	3.6 ± 0.7	4.8 ± 0.9	≤ 0.001
HDL (mmol/L)	1.3 ± 0.3	1.5 ± 0.4	0.003
LDL (mmol/L)	2.3 ± 0.6	3.3 ± 0.8	≤ 0.001
Cholesterol ratio	2.8 ± 0.6	3.3 ± 0.9	0.001

*Note:* Unless stated otherwise, data are presented as averages with standard deviations. Differences between groups were analyzed using unpaired *T*‐tests.

Abbreviations: BMI, body mass index; HDL, high‐density lipoprotein; HOMA‐IR, homeostatic model assessment of insulin resistance; LDL, low‐density lipoprotein.

### 3.2 Monocytes From Elderly Individuals Secrete Higher Lactate Levels Than From Young Adults

We first aimed to explore differences in monocyte immunometabolic signatures between young adults and elderly. Therefore, we performed principal component analysis (PCA) on metabolic and functional readouts from monocytes, including basal and activated (LPS, Pam3Cys, HKSA) glycolytic and oxidative metabolism, lactate secretion, phagocytosis, as well as mitochondrial oxidative capacities as determined by a mitochondrial stress test, and bead phagocytosis (Figure 1A). We observed a highly similar distribution of young adults and elderly along the first two principal components, explaining 48.8% of the variance in the dataset (Figure 1B). However, the PCA analysis revealed greater dispersion among the elderly individuals, indicating higher heterogeneity compared to the young adults. PC1 loadings showed a high contribution of secreted lactate in response to LPS, Pam3Cys, HKSA, and unstimulated—basal—lactate production (Figure 1C). Indeed, monocytes from the elderly secreted higher lactate levels at baseline and after stimulation compared to young adults (*p* ≤ 0.021) (Figure 1D). Noteworthy, no significant differences in lactate responses to the stimuli were observed when normalized to basal lactate secretion (Figure 1E).

### 3.3 Classification of Immune Fitness Based on Monocyte Lactate Secretion

**FIGURE 2:**
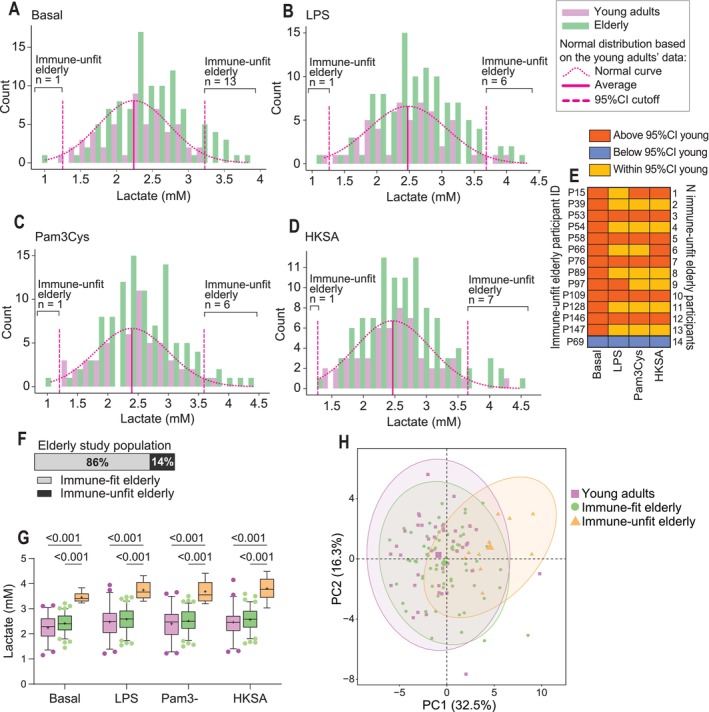
Classification of immune fitness by monocyte lactate secretion. (A‐D) Histograms for lactate levels for young adults (pink bars) and the elderly (green bars) with the normality curve and 95% confidence interval for the young adult population (pink dashed lines). Elderly with lactate levels outside the 95% CI range were classified as immune‐unfit. (E) Heatmap of immune immune‐unfit‐elderly showing the variable that resulted in the classification into the immune‐unfit elderly group. (F) The proportion of immune‐fit and immune‐unfit elderly in the total elderly study population. (G) Lactate secretion by monocytes upon 24 h exposure to control (RPMI) alone and in the presence of LPS (10 ng/mL), Pam3Cys (10 ng/mL), or HKSA (1 × 10^6^/mL) and stratified by immune‐fit and immune‐unfit elderly. (H) Principal component analysis on monocyte immunometabolic variables, including glycolytic activity, oxidative metabolic activity, cytokine secretion, and phagocytosis, but not lactate secretion, for immune‐fit and immune‐unfit elderly and young adults. Boxplots show median (middle line) and average (+). Whiskers cover 5%–95% of the data. Significance is reported in *p*‐values derived from two‐way ANOVA followed by Tukey's pairwise comparisons. 95% CI, 95% confidence interval; HKSA, heat‐killed *Staphylococcus aureus* ; LPS, lipopolysaccharide.

Our PCA analysis indicated monocyte lactate secretion as a variable that may contribute to the heterogeneity in monocyte immunometabolic signatures among the elderly. To identify the elderly with deviating monocyte lactate secretion levels compared to the young adults, we calculated the 95% confidence interval (CI) of basal lactate secretion from monocytes of the young adult study population. We observed that monocytes from 14 elderly participants secreted basal lactate levels outside the 95% CI of young adults (Figure 2A). Furthermore, from these 14 elderly participants, six elderly also had LPS and Pam3Cys‐induced lactate levels by monocytes above the 95% CI of the young adults, and seven elderly had HKSA‐induced lactate levels by monocytes above the 95% CI of young adults (Figure 2B–D). We hypothesized that the young adult population represents a healthy immune fitness; hence, we classified 14 elderly with poor immune fitness (Figure 2E), comprising 14% of the total elderly study population (Figure 2F). As expected, the lactate levels were significantly higher among these immune‐unfit compared to the immune‐fit elderly and young adults (*p* ≤ 0.001) (Figure 2G). Moreover, PCA analysis, including all monocyte functional and metabolic readouts, showed a divergence between the immune‐unfit elderly and the immune‐fit elderly and young adults (Figure 2H).

### 3.4 Monocytes From Immune‐Unfit Elderly Exhibit Metabolic Hyperactivity

**FIGURE 3:**
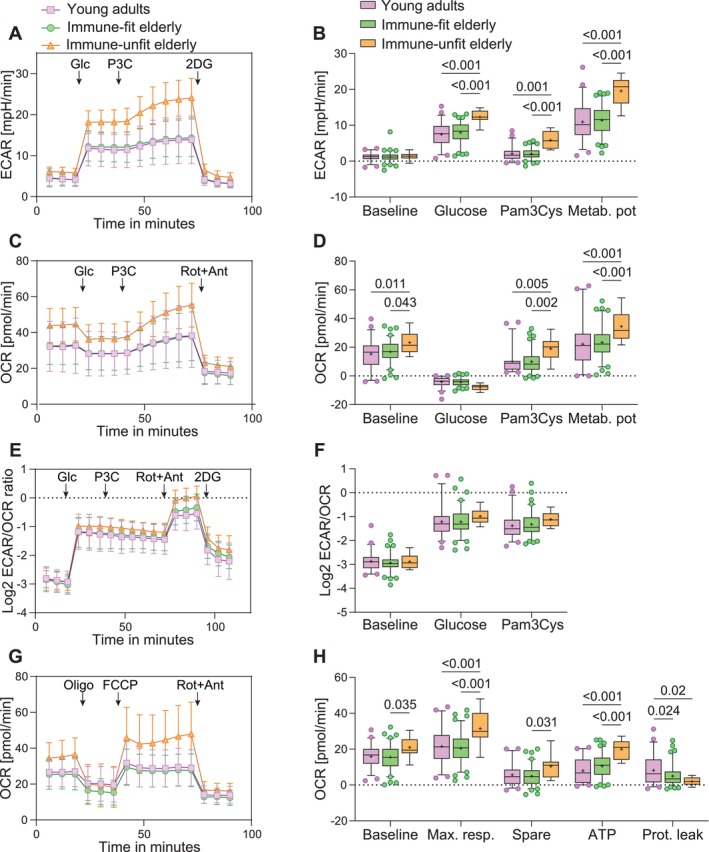
Monocyte metabolic activity in young adults and immune‐fit and immune‐unfit elderly. Freshly isolated monocytes were used for the seahorse assays. (A) ECAR time curves for the glucose and Pam3Cys challenge. (B) Time curve‐derived ECAR variables. The effect of glucose is relative to the average basal ECAR, and the effect of Pam3Cys is relative to the maximal ECAR upon glucose exposure. The metabolic potential refers to the highest ECAR measured during the assay. (C) OCR time curves for the glucose and Pam3Cys challenge. (D) Time curve‐derived OCR variables. The effect of glucose is relative to the average OCR at baseline, and the effect of Pam3Cys is relative to the average OCR upon glucose exposure. The metabolic potential refers to the highest OCR measured during the assay. (E) The ECAR‐to‐OCR (ECAR/OCR) ratio for metabolic dependency during the glucose and Pam3Cys challenge. A lower ECAR/OCR ratio represents lower glycolytic dependency. (F) ECAR/OCR variables are calculated as the average ECAR/OCR during baseline or in response to glucose and Pam3Cys. (G) Mitochondrial stress test (MST) and (H) derived MST variables. Line charts show averages and standard deviations for each time point. Boxplots show median (middle line) and average (+), and whiskers cover 5%–95% of the data. Significance is reported in *p*‐values derived from two‐way ANOVA followed by Tukey's pairwise comparisons. *N* young adults = 50, *N* immune‐fit elderly = 86, *N* immune‐unfit elderly = 12. ECAR, extracellular acidification rate; Max. resp., maximal respiration; Metab. Pot, metabolic potential; OCR, oxygen consumption rate; Prot. Leak, proton leak.

We further characterized differences in glycolytic and oxidative metabolic responses between young adults, immune‐fit and immune‐unfit elderly based on our stratification using lactate secretion of monocytes. Seahorse extracellular flux assays revealed that both glucose and TLR2 stimulation using Pam3Cys increased ECAR in young adults and the elderly (*p* ≤ 0.001) (Figure S1A). Comparison between young adults and the total group of elderly did not reveal significant differences, but we observed a trend toward higher ECAR in the elderly (Figure S1A,B). However, stratification of the elderly population by immune‐fitness showed significantly higher ECAR in response to glucose and Pam3Cys (*p* ≤ 0.001) and a higher ECAR metabolic potential—reflecting the highest ECAR measured during the assay—among the immune‐unfit elderly compared to immune‐fit elderly and young adults (*p* ≤ 0.001) (Figure 3A,B). Similarly, OCR was not different between young adults and the total group of elderly (Figure S1C,D). However, the stratified analysis showed significantly higher OCR at baseline and in response to Pam3Cys and a significantly higher OCR metabolic potential among the immune‐unfit elderly compared to immune‐fit elderly and young adults (*p* ≤ 0.04) (Figure 3C,D). The ECAR/OCR ratio—reflecting the relative metabolic dependency of monocytes—was not different between young adults and the elderly nor between immune‐unfit elderly, immune‐fit elderly, and young adults (Figures 3E,F and S1E,F).

To further explore differences in the mitochondrial capacity of monocytes between young adults and the elderly, we performed a mitochondrial stress test. We observed a significantly higher ATP‐linked OCR and reduced proton leak among monocytes from the elderly compared to young adults (*p* = 0.004; 0.007, respectively) (Figure S1G,H). However, ATP‐linked OCR was even higher among immune‐unfit elderly compared to immune‐fit elderly and young adults (*p* ≤ 0.001) (Figure 3G,H). Additionally, monocytes from immune‐unfit elderly showed significantly higher basal OCR, maximal respiration, and spare OCR compared to immune‐fit elderly and young adults (*p* ≤ 0.04) (Figure 3G,H). Hence, in addition to the higher oxidative metabolic responses to glucose and Pam3Cys in immune‐unfit elderly, these results show increased oxidative metabolic capacity and ATP production in monocytes from immune‐unfit elderly compared to immune‐fit elderly and young adults. These results indicate that the monocytes from immune‐unfit elderly are hypermetabolic.

### 3.5 Monocytes From Immune‐Unfit Elderly Show Elevated Cytokine Secretion

**FIGURE 4:**
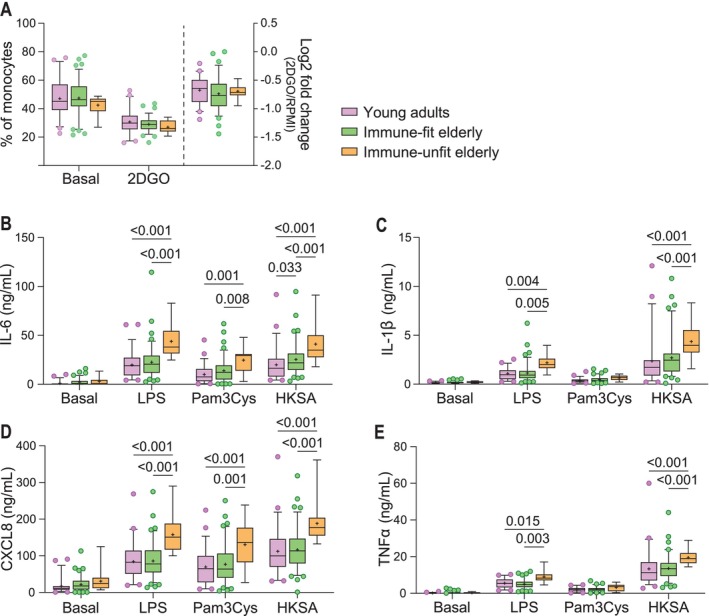
Monocyte functionality in young adults, immune‐fit, and immune‐unfit elderly. (A) Monocytes were pre‐incubated with RPMI‐only or supplemented with the metabolic inhibitors 2DG and oligomycin (2DGO) to inhibit phagocytosis. Subsequently, monocytes were exposed to fluorescent beads for 1.5 h, after which the intracellular beads were measured on the flow cytometer. Phagocytosis is expressed as the proportion of monocytes with intracellular beads. The effect of 2DGO on the proportion of monocytes performing phagocytosis is plotted on the right Y‐axis and calculated as the fold change of the 2DGO condition to RPMI‐only. *N* young adults = 50; *N* immune‐fit = 78; *N* immune‐unfit = 11. (B–E) Secreted cytokine levels by monocytes upon exposure to control (RPMI) alone or supplemented with LPS (10 ng/mL), Pam3Cys (10 ng/mL), or HKSA (1 × 10^6^/mL) for 24 h. Cytokine levels were measured in supernatants by ELISA. Monocyte cytokine samples: *N* = 52 young adults; *N* = 88 immune‐fit elderly; *N* = 13 immune‐unfit elderly. Boxplots show median (middle line) and average (+). Whiskers cover 5%–95% of the data. Significance is reported in *p*‐values derived from two‐way ANOVA followed by Tukey's pairwise comparisons.

We observed that immune fitness, as determined by monocyte basal lactate secretion, was linked with hypermetabolic responses. To explore if immune fitness was also associated with monocyte functionality, we measured phagocytosis and cytokine secretion. We did not observe differences in the proportion of monocytes that performed phagocytosis or the number of fluorescent beads taken up by the monocytes between the elderly and young adults (Figure S2A–D). Likewise, stratification of the elderly by immune fitness did not reveal differences in the proportion of monocytes that performed phagocytosis nor the number of beads taken up between the groups (Figures 4A, S2E–G).

Monocyte cytokine secretion was measured in supernatants after 24 h pathogenic activation. While we did not observe differences in secreted IL‐1β or CXCL8 in response to pathogenic stimuli between elderly and young adults, exposure to LPS, Pam3Cys, and HKSA led to a significantly higher IL‐6 secretion, and HKSA also significantly increased IL‐1β secretion by monocytes from the elderly compared to young adults (*p* ≤ 0.03) (Figure S3A–D). Stratification for immune fitness further showed significantly higher cytokine secretion by monocytes from immune‐unfit elderly compared to immune‐fit elderly and young adults, illustrated by significant increases in LPS and HKSA‐induced IL‐6, IL‐1β, CXCL8, and TNFα secretion (*p* ≤ 0.03) (Figure 4B–E). Additionally, Pam3Cys significantly increased IL‐6 and CXCL8 secretion by monocytes from immune‐unfit elderly compared to immune‐fit elderly and young adults (*p* ≤ 0.01) (Figure 4B,D). Hence, in addition to the observed hypermetabolic state, monocytes from immune‐unfit elderly also presented a hyperinflammatory state.

### 3.6 Immune‐Unfit Elderly Had Higher Circulating VEGFA and LDL‐c Levels Than Immune‐Fit Elderly

**FIGURE 5:**
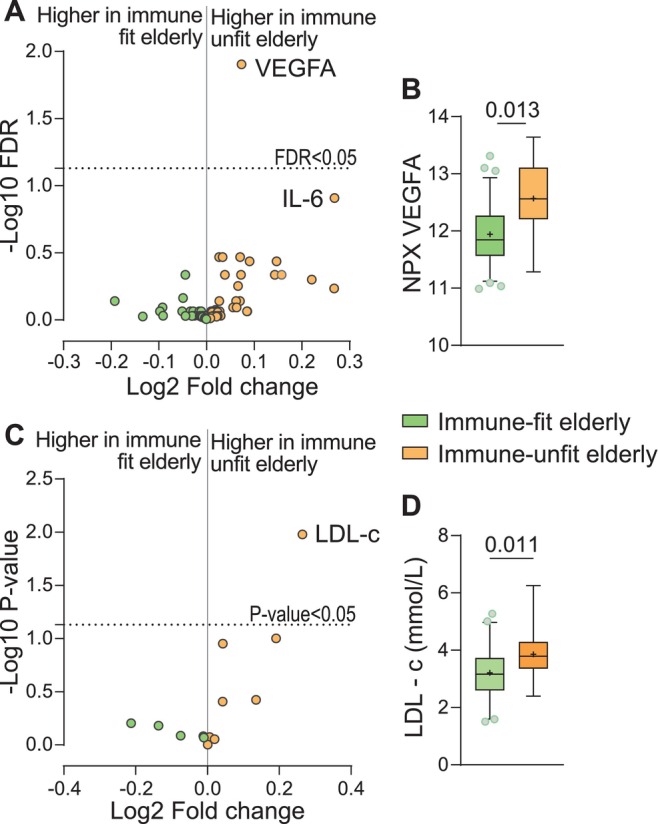
Immune‐unfit elderly had higher circulating levels of VEGFA and LDL‐c compared to immune‐fit elderly. Fasted blood samples were used for plasma proteomics (olink) and the measurements of clinical markers for metabolic health. (A) Volcano plot for fold change differences in circulating inflammatory proteins between immune‐fit and immune‐unfit elderly (B) and relative expression reported as normalized protein expression (NPX) of circulating VEGFA. *N* = 12 immune‐fit elderly and *N* = 77 immune‐unfit elderly. (C) Fold change differences in clinical parameters for body composition, glucose sensitivity, and cholesterol levels between immune‐fit and immune‐unfit elderly. (D) Circulating LDL‐c levels of *N* = 12 immune‐unfit and *N* = 88 immune‐fit elderly. Significance is derived from one‐way ANOVA. Boxplots show median (middle line) and average (+). Whiskers cover 5%–95% of the data.

The immune fitness classification represented hypermetabolic and hyperinflammatory monocytes—summarized as hyperactive monocytes—in the immune‐unfit elderly. To investigate whether immune fitness was associated with markers for systemic inflammation, we measured circulating inflammatory proteins in fasted plasma samples. We observed significantly elevated levels of circulating VEGFA, IL‐6, CD5, CDCP1, CXCL9, Flt3L, and MCP1 among immune‐unfit compared to immune‐fit elderly. Correcting for multiple testing highlighted VEGFA to be significantly increased in immune‐unfit elderly (FDR = 0.012) (Figures 5A,B, S4; Table S2). We then assessed clinical markers for metabolic health, including body composition, glucose sensitivity, and cholesterol levels between immune‐fit and immune‐unfit elderly (Table S3). Interestingly, we observed significantly higher LDL‐c levels among the immune‐unfit compared to the immune‐fit elderly (*p* = 0.011) (Figure 5C,D, Table S4). Hence, in addition to hyperactive monocytes, immune‐unfit elderly are characterized by elevated levels of circulating VEGFA and LDL‐c compared to immune‐fit elderly.

## 4 Discussion

This study provides a comprehensive overview of monocyte metabolism and functional responses of elderly individuals who were healthy as defined by the absence of metabolic complications and disease. We showed that monocytes of the elderly participants were highly similar to those of young adults based on metabolic and functional readouts. However, differences existed between both study populations, including a significantly lower mitochondrial proton leak and higher ATP‐linked respiration, lactate, and cytokine secretion by monocytes from the elderly compared to young adults. These differences were primarily driven by a subgroup of elderly individuals with monocyte‐secreted lactate levels higher than those of young adults, as determined by the 95% confidence interval. We observed that the monocytes from this subgroup with elevated lactate secretion levels had “hyperactive” phenotype, as evidenced by significantly elevated glycolytic and oxidative metabolic activity and capacity, along with higher cytokine secretion levels compared to monocytes from elderly individuals with similar monocyte lactate secretion levels as the young adult study population. Hence, we classified these elderly individuals with hyperactive monocytes as immune‐unfit. Intriguingly, significantly higher levels of circulating VEGFA and LDL‐c were found in immune‐unfit than in immune‐fit elderly. We propose monocyte lactate secretion as an early indicator for classifying immune‐unfit elderly with potentially an increased risk for cardiometabolic complications.

An important note is that the healthy elderly population is relatively underrepresented in the literature and that findings in frail, pre‐diseased, or sick elderly are easily generalized to the complete elderly population, leading to the conventional idea that aging leads to immune dysfunction. However, previous studies (Lissner et al. 2015; Shchukina et al. 2021; Qian et al. 2012; Yarbro and Pence 2019; Pence and Yarbro 2018, 2019; Grievink et al. 2022) and our research have shown that there is a high similarity in the magnitude and variation of monocyte functional and metabolic responses between young adults and healthy elderly individuals. These observations suggest that healthy aging may not necessarily coincide with immune dysfunction. Nonetheless, differences between healthy elderly and young study populations have been reported, but they are highly inconsistent among different studies. Our observation of increased pathogenic‐induced IL‐6 and IL‐1β secretion in the elderly is in line with previous observations on gene expression levels (Yarbro and Pence 2019). However, previous studies also reported elevated secretion of TNFα and CXCL8 by monocytes from healthy elderly, while our results showed no differences (Hearps et al. 2012; Cao et al. 2022; Qian et al. 2012; Ong et al. 2018). In contrast, others have shown reduced cytokine secretion by monocytes from healthy elderly compared to young adults, including IL‐6, TNFα, and CXCL8 (Cao et al. 2022; Grievink et al. 2022; Nyugen et al. 2010). Monocyte metabolic responses have also previously been studied. Opposite to our results, a smaller study including eight healthy elderly individuals reported lower monocyte maximal respiratory capacity compared to young individuals (Pence and Yarbro 2018). Thus, while our findings highlight a strong similarity in monocyte function and metabolism between young adults and healthy elderly individuals, subtle but notable differences suggest that within a healthy elderly population, there may be individuals with early signs of immune dysfunction.

The identification of healthy individuals with early signs of immune dysfunction requires in‐depth immunophenotyping to gain comprehensive insight into immune cell fitness. Our study filled this gap by incorporating a detailed analysis of monocyte metabolic signature, in addition to functional responses, in young adults and healthy elderly individuals. It was previously shown that individuals can be classified as high or low pathogenic‐induced cytokine responders based on lactate secretion by PBMCs (Vrieling et al. 2022), and given its central role in the regulation of intracellular metabolism (Fang et al. 2024), we anticipated that lactate could potentially serve as an indicator for both the metabolism and functionality of monocytes. Importantly, we made the assumption that young adults represent healthy immune responses of monocytes, and yet, we classified the elderly individuals with monocyte lactate secretion levels deviating from this young, healthy immune‐fit population as immune‐unfit. For this classification, we relied on basal lactate secretion levels, as we consider these to better reflect the in vivo situation compared to ex vivo pathogenic challenges. Notably, some immune‐unfit elderly had ex vivo pathogenic stimulated lactate secretion levels that were similar to those of young adults depending on the type of stimulus. This discrepancy in fit or unfit classification based on basal and stimulated lactate levels may be explained by stimulus‐specific tolerance as it was previously shown that aging differentially impacts expression and signaling of different pathogen recognition receptors (Shaw et al. 2011). Within our elderly study population, intraindividual variability may exist related to stimulus‐specific tolerance.

Our study's intensive characterization of monocyte metabolic and functional responses enabled us to evaluate innate immune fitness across a broad spectrum of monocyte characteristics, including mitochondrial capacity, oxidative and glycolytic responses to glucose and Pam3Cys, phagocytic activity, and pathogenic‐induced cytokine release. Since all these readouts—except for phagocytic activity—were significantly higher among the immune‐unfit elderly compared to the immune‐fit elderly, we identified a subgroup of elderly who are immunologically divergent from the majority of the elderly in our study population.

It can be argued that the hyperactivity observed in the immune‐unfit elderly actually reflects “unfit” responses, and it is currently unknown whether this will contribute to the onset of age‐related complications in the long term. Positive CD14 selection of monocytes may have biased our results, as CD14 is involved in inflammatory and metabolic regulation (Na et al. 2023). The hyperactive status of monocytes could also represent a compensatory mechanism, as aging is related to the loss of epidermal and mucosal epithelial barrier function (de Vries et al. 2022; Wang et al. 2020) requiring a higher level of activity of the immune system. On the other hand, elevated levels of inflammation have also been related to the onset of age‐related complications (Furman et al. 2019), and the monocyte hyperactivity of immune‐unfit elderly fits with the concepts of chronic inflammation. This supports a model in which the hyperactive profile of monocytes represents a dysfunctional innate immune system. Hence, the proposed novel immune fitness classification has the potential to identify early innate immune dysfunction among healthy individuals, and could therefore be used to identify individuals at risk for unhealthy aging. Follow‐up studies should serve to reveal a potential mechanism to explain the hypermetabolic state of monocytes.

One notable observation in our study was the elevated oxidative metabolism in monocytes from immune‐unfit elderly individuals compared to the immune‐fit elderly, while conventional ideas on aging and inflammaging often describe mitochondrial dysfunction, characterized by reduced oxidative metabolism (van Beek et al. 2019; Lima et al. 2022). These conventional concepts were previously supported by studies among healthy elderly individuals, showing that monocytes express lower levels of mitochondrial genes, including those coding for electron transport chain proteins, suggesting reduced mitochondrial oxidative metabolism (Shchukina et al. 2021), and maximal respiratory capacity (Pence and Yarbro 2018). Hence, the increased Pam3Cys‐induced oxidative metabolic response, as well as the higher spare, maximal, and ATP‐linked respiration in monocytes from immune‐unfit elderly compared to immune‐fit elderly individuals in our study, contrasts these previous observations. In line with our study, recent research showed that B‐cells from six healthy elderly individuals exhibited a higher maximal respiratory capacity than those from young individuals (Romero et al. 2024), and T‐cells from healthy elderly had a lower proton leak and increased ATP‐linked oxidative metabolism (Yanes et al. 2019). Additionally, a trend toward higher LPS‐induced oxidative metabolism in monocytes among nine healthy elderly has also previously been observed (Pence and Yarbro 2019). Based on these observations, it could be postulated that given the dynamic nature of aging, mitochondrial hyperactivity precedes the commonly described decline in mitochondrial function. This phenomenon of heightened activity followed by impaired functionality has been previously described in the context of various diseases, such as beta‐cell failure in the progression of insulin resistance (Hudish et al. 2019). Hence, we speculate that mitochondrial hyperactivity, as part of monocyte hyperactivity, may serve as an early indicator of immune dysfunction before metabolic complications are perceptible.

We found that immune‐unfit elderly had elevated levels of circulating VEGFA—also known as VEGF—and LDL‐c, which are biomarkers for increased cardiovascular risk (Abdullah et al. 2018; Heeschen et al. 2003). These elevated circulating levels of VEGF and LDL‐c in the immune‐unfit elderly may either be a cause or a consequence of monocyte hyperactivity. While elevated LDL‐c levels in elderly individuals are related to cardiovascular complications and increased mortality, the role of VEGF in aging is controversial (Abdullah et al. 2018; Dabravolski et al. 2022). In healthy aging, VEGF can promote tissue perfusion and oxygenation and may be protective against organ aging (Keren et al. 2022; Grunewald et al. 2021). VEGF levels are often elevated among individuals with cardiovascular complications (Smith et al. 2015), but VEGF‐mediated angiogenic effects are considered harmful in these patients due to the destructive effects on plaque stability (Dabravolski et al. 2022). Notably, it has been shown that VEGF promotes monocyte differentiation into pro‐inflammatory macrophages (Hu et al. 2021), and it could, therefore, be speculated that VEGF may contribute to a pro‐inflammatory signature in circulating monocytes that is reflected by the hyperactive immunometabolic signatures as we observed in the immune‐unfit elderly. In the case of LDL‐c, elevated circulating levels were previously associated with pro‐inflammatory monocytes in patients with familial hypercholesterolemia (Stiekema et al. 2021). Furthermore, monocyte metabolic hyperactivity was previously shown in adults and elderly with coronary artery disease (Zeisbrich et al. 2018), and inflammation is a well‐known driver of atherosclerosis progression (Jukema et al. 2019). Taken together, circulating VEGF and LDL‐c levels and monocyte hyperactivity may bidirectionally affect one another.

In conclusion, we extensively characterized circulating monocytes of healthy elderly and created a comprehensive understanding of their metabolic and functional behavior. An important observation was the high similarity in monocyte metabolic and functional responses between the elderly and young adults. However, using lactate secretion levels by monocytes from young adults as a reference, we classified a group of elderly—the immune‐unfit group—who presented monocyte hyperactivity, illustrated by significantly higher metabolic and functional responses in monocytes compared to the remainder of the elderly study population, the immune‐fit group. Given the previously established links between excessive inflammation and metabolic complications (Baechle et al. 2023), and our observation of elevated VEGFA and LDL‐c levels among the immune‐unfit elderly that may relate to cardiovascular risk in our study population (Abdullah et al. 2018; Smith et al. 2015), we anticipate that the immune fitness classification may hold clinical and translational relevance, and could aid the identification of healthy elderly at risk for unhealthy aging. The immune‐fitness classification may also aid the design and assignment of personalized anti‐inflammatory prevention strategies, for instance focused at reducing glycolytic metabolism in immune cells through nutritional and lifestyle adjustments (Bulut et al. 2020; Cadar et al. 2023). Hence, longitudinal studies would be highly valuable in studying the consequences of the immune‐unfit classification, such as the onset, progression, and severity of age‐related metabolic complications, and in facilitating the establishment of monocyte lactate secretion as a biomarker of immunological health.

## Author Contributions

L.S., G.G., J.A.D., L.A.A., and R.S. designed and organised the study. L.S., F.V., J.J., and H.J.P.Z. performed the study and collected data. L.S., F.V., and T.M.H. performed statistical analysis. L.S., L.A.A., and R.S. wrote the manuscript, and all other authors read and approved the manuscript. L.A.A. and R.S. are the guarantors of this work.

## Conflicts of Interest

The authors declare no conflicts of interest.

## Supporting information


**Figure S1:** acel70220‐sup‐0001‐FigureS1.ai.

## Data Availability

The data that support the findings of this study are available from the corresponding author upon reasonable request.
